# Peripheral Mechanisms of Neuropathic Pain—The Role of Neuronal and Non-Neuronal Interactions and Their Implications for Topical Treatment of Neuropathic Pain

**DOI:** 10.3390/ph14020077

**Published:** 2021-01-20

**Authors:** Magdalena Kocot-Kępska, Renata Zajączkowska, Joanna Mika, Jerzy Wordliczek, Jan Dobrogowski, Anna Przeklasa-Muszyńska

**Affiliations:** 1 Department of Pain Research and Treatment, Medical College, Jagiellonian University, 31-531 Krakow, Poland; jan.dobrogowski@uj.edu.pl (J.D.); a.przeklasa-muszynska@uj.edu.pl (A.P.-M.); 2 Department of Interdisciplinary Intensive Care, Medical College, Jagiellonian University, 30-688 Krakow, Poland; renata.zajaczkowska@uj.edu.pl (R.Z.); j.wordliczek@uj.edu.pl (J.W.); 3 Maj Institute of Pharmacology, Polish Academy of Sciences, Department of Pain Pharmacology, 31-343 Krakow, Poland; joamika@if-pan.krakow.pl

**Keywords:** neuropathic pain, topical, ion channels, peripheral sensitization, pain management

## Abstract

Neuropathic pain in humans arises as a consequence of injury or disease of somatosensory nervous system at peripheral or central level. Peripheral neuropathic pain is more common than central neuropathic pain, and is supposed to result from peripheral mechanisms, following nerve injury. The animal models of neuropathic pain show extensive functional and structural changes occurring in neuronal and non-neuronal cells in response to peripheral nerve injury. These pathological changes following damage lead to peripheral sensitization development, and subsequently to central sensitization initiation with spinal and supraspinal mechanism involved. The aim of this narrative review paper is to discuss the mechanisms engaged in peripheral neuropathic pain generation and maintenance, with special focus on the role of glial, immune, and epithelial cells in peripheral nociception. Based on the preclinical and clinical studies, interactions between neuronal and non-neuronal cells have been described, pointing out at the molecular/cellular underlying mechanisms of neuropathic pain, which might be potentially targeted by topical treatments in clinical practice. The modulation of the complex neuro-immuno-cutaneous interactions in the periphery represents a strategy for the development of new topical analgesics and their utilization in clinical settings.

## 1. Introduction

Neuropathic pain is defined by the International Association for the Study of Pain as pain caused by a lesion or disease of the somatosensory nervous system [1]. Neuropathic pain (NP) is not a particular disease, but the clinical condition that is caused by a variety of different diseases and lesions, injuring the nervous system at peripheral or central level, resulting in peripheral NP or central NP, respectively. The nerve damage and subsequent functional and structural neuroplasticity in sensory and autonomic nervous system, may become pathological and maladaptive in certain percentage of patients, and the risk of maladaptation depends on biological, genetic, demographic, and psychosocial factors [2]. The nerve damage induces extensive response in immune system as well, resulting in close interactions between nervous and immune system, and finally neuroinflammation. The functional and structural neuroplasticity and complex neuro-immune interactions result in inappropriate signaling from periphery, inappropriate modulation, and disturbed central processing of pain. In clinical practice, chronic neuropathic pain may be thus considered as neuro-immunological disorder with multiple neuronal and non-neuronal mechanism involved, either in periphery or centrally [3,4,5]. In humans, NP features may vary according to the location and character of the nervous system lesion, but it has been suggested that a more peripheral lesion induces more localized signs and symptoms of NP [6]. In 2010, the first definition of localized neuropathic pain was proposed, aiming at description of a special type of NP, which is caused primarily by the injury of peripheral nervous system. Localized neuropathic pain (LNP) is a type of neuropathic pain that is characterized by consistent and circumscribed area(s) of maximum pain, associated with negative or positive sensory signs and/or spontaneous symptoms characteristic of neuropathic pain [7], and is felt in superficial tissues [6]. In patients with LNP, complex peripheral mechanisms following nerve injury are suggested to generate and maintain pain and sensory abnormalities [8]. Thus, there is a rationale for use of topical analgesics, acting locally at peripheral level, which is in line with current principles in pain medicine pointing out at the need of personalized and mechanism-based approach to pain management [9]. Topically applied analgesics are supposed to target the underlying molecular/cellular mechanisms in the periphery only, without systemic mechanisms and site of action. The preclinical data support this idea, but, in clinical practice, only a few of peripheral mechanisms of NP are currently addressed [10,11,12,13,14] This review of literature is aimed at presenting the available evidence from preclinical and clinical studies on the peripheral mechanisms of NP with special focus on interactions between neuronal and non-neuronal cells, the molecular targets for topical analgesics, and clinical implications for topical administration in NP management. The current knowledge on complex mechanism of NP and possible molecular targets for analgesics administered by topical routes is crucial for health care professionals dealing with patients suffering from NP.

## 2. Peripheral Mechanisms of NP

### 2.1. The Role of Neuronal Cells in Peripheral Mechanisms of NP

Several hypotheses have been proposed to explain the complex processes of generation, maintenance of NP, and underlying mechanisms, but the pathophysiology of NP remains unclear, despite the huge progress made to date. It is known that the peripheral sensory neurons with the cell bodies located in the dorsal root ganglia conduct nociceptive information, which enters the spinal cord dorsal horn and then from the spinal projection is conveyed to supraspinal structures (such as the brainstem, thalamus, somatosensory cortex, insular cortex and anterior cingulate cortex) via ascending pathways [4,15,16,17]. In humans, peripheral somatosensory nervous system may become injured at several levels. There are multiple routes to its damage, including mechanical, thermal, chemical, and infectious factors. Peripheral nerve endings of pain-processing unmyelinated C fibres and thinly myelinated Aδ fibres may become injured by metabolic damage, toxins, medications, cytokines, and other inflammatory mediators. The axon may be damaged by trauma, compression, hypoxia, inflammation, overload, and chemical factors, and finally neurons in the DRG (dorsal root ganglion) may be exposed to several chemical and mechanical factors as well. Axonal and DRG damage may subsequently induce pathological and pain-promoting changes in peripheral autonomic nervous system [18,19]. In humans, the data regarding pathophysiologic mechanisms initiated after nerve injury is scarce. However, the common features of NP including spontaneous or evoked, burning, shooting pain, allodynia, hyperalgesia, or sensory loss, suggest likelihood of shared underlying pathology [17]. The more detailed data on NP pathophysiology comes from experimental animal models of NP. Peripheral mechanisms have been extensively studied in several animal models, such as spared nerve injury (SNI), chronic constriction injury (CCI), spinal nerve ligation (SNL), and specific disease-related neuropathies such as rodent models of diabetes, chemotherapy, herpes zoster, HIV (human immunodeficiency virus) induced peripheral neuropathy [20,21]. In animal models of NP after a peripheral nerve injury, independently of its character, extensive functional, structural, and molecular changes have been observed, either in damaged or neighbouring undamaged nociceptive neurons (Aδ, C), or either in peripheral nerve endings, along the axon or in DRG neurons [22]:

- peripheral fibre density changes—partial loss of peripheral innervation due to physical injury, chemical or metabolic neurotoxicity [23,24,25];
- fibre degeneration—axonal loss due to Wallerian degeneration (self-determined process leading to cytoskeletal destabilization and fragmentation) [25,26,27,28];
- peripheral sensitization—hyperexcitability of sensory neurons due to lowered threshold and augmented response to suprathreshold stimuli, caused by peripheral nerve or tissue injury, inflammation and subsequent release of pro-nociceptive mediators from mast cells, macrophages and from neighbouring nerve terminals, such as prostaglandins, bradykinin, histamine, serotonin, SP (substance P), extracellular ATP (adenosine triphosphate), protons, cytokines, chemokines, growth factors, peptides, acting on corresponding receptors, ion channels or altering their sensitivity to stimuli [8,29,30];
- ectopic firing in peripheral nerve endings and in DRG neurons—ectopic discharge begins in Aδ fibres within hours after injury and within several days or weeks in C fibres [31,32,33,34]; the main generator of ectopic activity are hyperpolarization-activated and cyclic nucleotide-gated (HCN) channels, belonging to the voltage-gated potassium (Kv) channels [35]; in human altered firing and ectopic activity in peripheral neurons was observed in patients holding a mutation in gene coding Nav1.7 [36];
- alterations in channel expression and composition in peripheral nerve endings, along the axon and in DRG—peripheral input via intracellular second messengers alters gene expressions, resulting in increase in protein expression of Nav (voltage-gated sodium channels), VGCC (voltage-gated calcium channels), TLR4 (toll-like receptors 4), TRP (transient receptor potential channels), α1-AR (α1 adrenergic receptors), ASIC (acid-sensing ion channels), decrease in protein expression of Kv (voltage-gated potassium channels) [37,38,39,40];
- synapse properties and locations of spinal terminals—sprouting of Aβ fibres in spinal dorsal horn laminae [41,42];
- involvement of autonomic system—upregulation of α1-AR and enhanced adrenergic sensitivity at the injury site and in DRG neurons, sympathetic fibres sprouting in the periphery and in DRG [43,44,45,46].

It is worth mentioning that the pathological neuroplasticity after peripheral nerve injury in preclinical settings is seen not only in nociceptive Aδ and C fibres [8,47,48,49], but in the population of Aβ fibres as well. Aβ fibres respond normally to innocuous mechanical stimuli and in physiology are not involved directly in nociception. In animal models of NP, Aβ fibres exhibited enhanced excitability, spontaneous activity, differences in action potential configuration and conduction velocity compared with control animals [50]. The data shows as well abnormal axonal sprouting of myelinated Aβ axons in the spinal dorsal horn. The peripheral receptive fields of Aβ neurons are more excitable, which in summary contributes to the generation and maintenance of the peripheral, central sensitization and NP [42,51,52]. The pathology in tactile Aβ neurons, resulting in nociceptive responses to normally innocuous cutaneous stimuli, is observed in humans with NP and clinically refers to allodynia and spontaneous pain following peripheral nerve injury [8,53,54]. 

**Figure 1:**
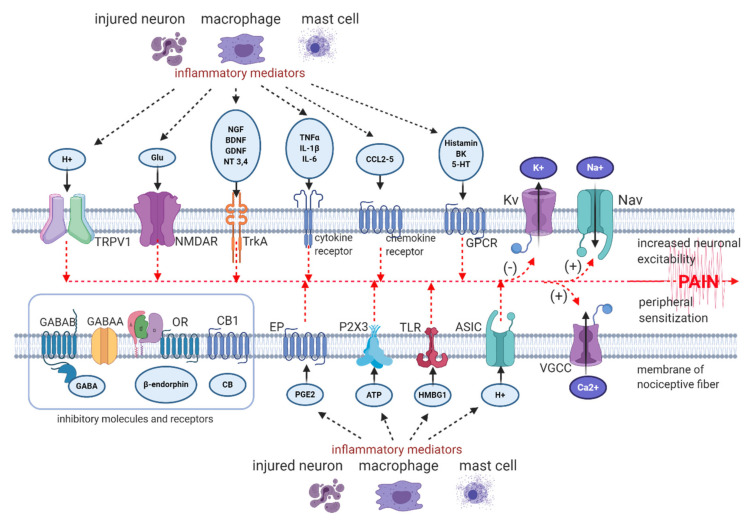
Neuronal ion channels, receptors and their ligands involved in peripheral neuroinflammation and sensitization. (−) Downregulation of Kv and K+ currents, (+) upregulation of Nav, VGCC, and Na^+^ and Ca^2+^ currents, respectively [8,22,29,30,31,32,33,34,35,36,37,38,39,40]. Abbreviations: Nav—voltage-gated sodium channel, TRPV1—transient receptor potential vanilloid 1 channel, VGCC—voltage-gated calcium channel, Glu—glutamate, H^+^—hydrogen proton, NMDAR—N-methyl-D-aspartate receptor, ASIC—acid sensing ion channel, TLR—toll-like receptor, P2X3—P2X purinoceptor 3, PGE2—prostaglandin E2, EP—prostaglandin E2 receptor, GABA—gamma-aminobutyric acid, GABAAR—gamma-aminobutyric acid receptor A, GABABR—gamma-aminobutyric acid receptor B, Kv—voltage-gated potassium channel, OR—opioid receptor, CB—cannabinoid, CB1—cannabinoid receptor type 1, HMBG1—high mobility group box 1 protein, TNFα—tumour necrosis factor α, IL-1β—interleukin 1β, IL-6—interleukin 6, CCL—CC-chemokine ligand, ATP—adenosine triphosphate, NGF—nerve growth factor, BDNF—brain-derived neurotrophic factor, GDNF—glial-derived neurotrophic factor, NT 3,4—neurotrophin 3 and 4, Na^+^—sodium ion, Ca^2+^—calcium ion, K^+^—potassium ion, BK—bradykinin, 5-HT—serotonin, GPCR—G protein-coupled receptor. *Created with BioRender.com.*

The molecular processes involved in neuroinflammation and peripheral sensitization are presented on Figure 1 [8,22,29,30,31,32,33,34,35,36,37,38,39,40].

### 2.2. Role of Glial Activation in Peripheral Mechanisms of NP 

In animal models of NP after a nerve injury, extensive functional, structural, and molecular changes, parallel to that observed in neurons, have been seen in glial cells as well. Injury of peripheral nerve leads to significant activation of peripheral glia including Schwann cells in the nerve, satellite glial cells in DRG, and central glial cells including microglia and astrocytes in the spinal cord and brain. The involvement of other macroglia cells such as radial cells and oligodendrocytes in the nociceptive transmission has not been established to date [55,56]. After peripheral nerve injury, the activation of astrocytes in CNS (central nervous system) occurs about four days after microglial activation and persists until 12 weeks after damage, thus suggesting being involved in the persistence of pain [56]. Therefore, it is so important to silence the escalation of neuroimmune peripheral changes at an early stage by topical drug administration, which may reduce the risk of the development of central pain hypersensitivity. The glial activation includes proliferation, morphological changes, increased or de novo expression of cell membrane markers or receptors, and the synthesis of numerous mediators. Glial activation is a defensive mechanism; however, it may malfunction after a nerve injury, leading to pain generation and its maintenance [55,56]. Schwann cells are most abundant glial cells in peripheral nervous system. They physically support long axons and produce numerous growth factors to nourish and myelinate axons, such as NGF (nerve growth factor), BDNF (brain-derived neurotrophic factor), GDNF (glial-derived neurotrophic factor), NT3 (neurotrophin 3) and NT4 (neurotrophin 4) [57]. Mounting evidence from preclinical studies suggests a key role of Schwann cells in peripheral NP states. After peripheral nerve injury, Schwann cells become activated, change their phenotype, proliferate, migrate, and release growth factors and other molecules promoting nerve regeneration. The role of growth factors in generation of NP has been confirmed in preclinical model, where the pain hypersensitivity resulted from release of BDNF from Schwann cells. The role of BDNF in NP has been further confirmed in BDNF-knockout mice, which displayed reduced pain behaviour compared to wild-type mice [58]. In NP conditions, multiple receptors and ion channels are expressed and upregulated on Schwann cells membrane: purinergic receptors, TLR, TRPA1 (transient receptor potential ankyrin 1), GABABR (gamma-aminobutyric acid receptor B), Ach (acetylcholine) receptors just to mention the most important in nociception. Exogenic molecules released from injured tissue, immune cells and neurons bind to corresponding receptors on Schwann cell membrane and via intracellular signalling promote release of growth factors, cytokines, and chemokines. The activation of Schwann cells results in the release of both proinflammatory cytokines (TNFα—tumour necrosis factor α, interleukin IL-1β, IL-6) and anti-inflammatory cytokines (IL-10, Epo (erythropoietin)). Moreover, activated Schwann cells produce chemokines (CCL2—CC-chemokine ligand 2), growth factors (NGF, BDNF, GDNF, NT3, NT4), and messenger molecules (ATP), which together with cytokines can modulate nociceptive input [59]. Modulation of NP involve recruitment of immune cells to the site of injury as well. In model of sciatic nerve injury, axonal damage stimulates primary Schwann cells via TLR3 activation to release macrophage-recruiting chemokines (CC-chemokine ligands CCL2, CCL4 and CCL5) and subsequent macrophage recruitment to injured nerves [60]. Proinflammatory cytokines, mainly TNFα, produced by activated Schwann cells and macrophages contribute to axonal damage and enhanced nociceptor activity. TNFα can alter the sensitivity of neurons to neurotransmitters via either increased activity and overexpression of neuronal ion channels, such as TRPV1 (transient receptor potential vanilloid type 1), AMPAR (α-amino-3-hydroxy-5-methyl-4-isoxazolepropionic acid receptor), VGCC, NMDAR (N-methyl-D-aspartate receptor), or down regulation of neuronal inhibitory GABA receptors [61]. Numerous receptors expressed and mediators released by Schwann cells in response to nerve injury, and data from preclinical studies confirmed direct interaction loop between activated neurons, Schwann cells, keratinocytes, and immune cells in the site of injury. Thereby, Schwann cells play a key role in regulating neuroinflammation and peripheral sensitization in NP conditions [55,56].

### 2.3. Role of Immunocompetent Cells in Peripheral Mechanisms of NP

Preclinical models of NP provide evidence for a substantial role of interactions between the nervous and immune system, resulting in neuroinflammation, altered sensory processing and evoked thermal and tactile hypersensitivity, but at the same time in damage repair as well [62] Among the immune cells, tissue-resident and recruited macrophages are supposed to play a key role in regulating neuroinflammation and peripheral NP [5,63,64]. Macrophages are plastic and may play opposite roles: proinflammatory M1 macrophages expressing proinflammatory cytokines (TNFα, IL-1β, IL-6, IL-18), chemokines (CCL2-5) and toll-like receptors (TLR4), releasing ROS (reactive oxygen species), and anti-inflammatory M2 macrophages expressing anti-inflammatory cytokine IL-10 and chemokines CCL18, CCL22 and CCL24. In NP states, prolonged activation of M1 macrophages has been observed, which probably results from activation of TLR4 expressed on their cell membrane. In experimental animals, treatments directed at M1 macrophages reduced inflammation, pain behaviour in nerve injury and chemotherapy induced pain models [65,66].

Macrophages express α1-AR as well, and in preclinical studies activation of α1-AR by phenylephrine resulted in increased production of IL-1β in human monocytes and macrophages. On a cellular level, AR can modify cytokine production by macrophages by activation of TLR [67]. After injury, damaged neurons, Schwann cells, tissue-resident macrophages produce various cytokines, chemokines, and other signalling proteins, which recruit immunocompetent cells (macrophages, neutrophils, lymphocytes) to the injured site [68]. For example, in a model of a sciatic nerve crush injury, axonal damage stimulates Schwann cells to release macrophage-recruiting chemokines (CCL2, CCL4 and CCL5), resulting in macrophage recruitment to injured nerves [60] At the injured site, immune cells and Schwann cells release numerous proinflammatory cytokines (TNFα, IL-1β, IL-6), and chemokines (CCL2, CCL3, CCL4), acting on upregulated cytokine and chemokine receptors in peripheral nerve endings and DRG. The proinflammatory molecules via their receptors change the excitability, ion currents and second messenger systems of peripheral neurons, leading to peripheral sensitization and hyperalgesia. The cytokines may sensitize ion channels responsible for the transduction of stimuli (TRP—TRPA1, TRPV1, TRPV4) or alter the function of voltage-gated ion channels responsible for the regulation of the membrane potential (Nav, VGCC, Kv) [5,69,70,71,72]. Proinflammatory cytokines released from immune cells may increase the expression of alfa1-AR in keratinocytes and neurons, leading to local hyperalgesia in animal model of burn [73,74]. The expression of α1-AR on monocytes, macrophages, keratinocytes, and neuronal cells confirms that mediators of the neuroendocrine system (e.g., catecholamines) may modify neuroinflammatory responses. Further, it confirms the role of sympathetic system in NP generation and maintenance, suggesting a possible role of treatments addressing upregulated α1-AR in NP management [67]. Peripheral nerve injury induces Schwann cells and macrophages to release arachidonic acid and synthesis of its derivates, mainly prostaglandins (PGs) via COX-2 (cyclooxygenase-2) induction by cytokines. After an injury, PGs may be synthetized not only in recruited immune cells, but in neuronal cells as well. PGs regulate the function of peripheral sensory nerves in paracrine and autocrine manners in animal models on NP [75]. PGE2 (prostaglandin E2) via its EP receptor (prostaglandin E2 receptor), expressed in neuronal membrane, can modulate the excitability of peripheral nerve endings. PGE2 sensitizes ion channels and receptors (TRPV1, Nav1.7,1.8,1.9, VGCC, P2X3 (P2X purinoceptor 3)) and down regulates the Kv, which results in enhanced Na currents and Ca2+ influx, reduced K+ currents, peripheral hyperexcitability and increased neurotransmitter release at the spinal level [76]. At the injury site, recruited macrophages interact with tissue-resident cells such as macrophages, mast cells and dendritic cells by releasing mediators such as CCL2, TNFα, IL-1α, IL-1β, and PGE, among others. These mediators act on corresponding receptors, expressed by immune cells and stimulate the release of cascade of pro- and anti-inflammatory mediators, simultaneously enhancing further infiltration of immune cells to the site of injury. These interactions elicit long-lasting neuroinflammation and maintain the NP [5,77]. It is worth mentioning that immune cells express numerous receptors for ligands with potential inhibitory (anti-inflammatory or immunosuppressive) mechanisms of action. Macrophages, mast cells, microglia and other immune cells express CB2 (cannabinoid receptor type 2) receptor and OR (opioid receptor). In preclinical trials, it has been observed that agonists of CB2 receptor exert an anti-inflammatory effect, which might be potentially beneficial in chronic pain states [78]. In turn, in preclinical and clinical settings OR agonists such as morphine or fentanyl exerts immunosuppressive effect, resulting in reduction of either macrophage numbers or production of macrophage proinflammatory cytokines [79] Potentially, immune cells involved in peripheral neurogenic inflammation and their receptors may be target for topically applied cannabinoids or opioids [80,81,82]. Immune cells express GABA receptors and GABA as well. GABA agonist i.e., baclofen exerts an antipruritic mechanism in chronic dermatitis, but the role of interaction GABA receptor and its agonist in immune cells in NP is not confirmed [83,84]. Taken together, immunocompetent cells through their complex communication with Schwann cells, neurons and keratinocytes can induce local chronic neuroinflammation, ongoing keratinocyte, and peripheral nerve endings stimulation, which results in peripheral sensitization and NP. Potentially, immunocompetent cells involved in peripheral neuroinflammation and their receptors may be a target for topical treatments, acting on specific receptors, ion channels or enzymes (i.e., TLR, α1-AR, COX-2, GABAR, CB, OR).

### 2.4. The Role of Skin Cells in Peripheral Mechanisms of NP

The idea of the neuro-immuno-cutaneous system (NICS), including peripheral sensory neurons, immune cells, and cutaneous cells, is becoming more recognized in clinical practice. The skin, besides being homeostatic and immunological barrier, acts as a sensory organ as well, since NICS is responsible for cutaneous sensations, such as touch, pressure, temperature, and pain. Sensory neurons are located in all layers of the skin: in the epidermis, dermis, and hypodermis. In the epidermis, the outermost layer of the skin, sensory nerve endings interact with other skin cells (keratinocytes and immune cells) in several ways, either via neurotransmitters, neuropeptides, and cytokines, or via membrane associations. The anatomical and functional interactions between neuronal and non-neuronal cells in the skin are contributing to nociception, neuronal hyperexcitability and peripheral sensitization [85,86]. The keratinocytes, which constitute 90% of epidermis cells, are one of the main targets of topically applied analgesics. The preclinical studies showed stimulation of keratinocytes alone to be sufficient to induce neuronal hyperexcitability and pain behaviour in animals [87,88]. According to preclinical data, keratinocytes express several receptors and ion channels, playing role in nociception, such as Nav (Nav 1.1,1.2, 1.5,1.6,1.7,1.8), TRP (TRPV1-4), neurokinin 1 receptor (NK1-receptor, NK1R), TLR, interleukin receptors, α1-AR, endothelin1 receptors (ET1), calcitonin receptor-like receptor (CRLR), CB receptors, OR, NOP (nociceptin-orphanin opioid peptide) receptors (NOP-R), VGCC, NMDAR, and GABAR to mention a few [89,90,91,92,93,94,95,96]. The role of keratinocyte receptors and their overexpression in nociception has been confirmed in clinical observations. In patients with small fibre neuropathy a statistically significant increase of TRPV1 expression on epidermal keratinocytes was reported [97] The skin biopsies from patients with complex regional pain syndrome (CRPS) or postherpetic neuralgia (PHN) were analysed, and the samples exhibited Nav1.1, Nav1.2, Nav1.5, Nav1.6, and Nav1.7 and Nav1.8 immunolabeling, which was not present in normal skin [91]. Besides expressing numerous receptors and ion channels, in physiological conditions, keratinocytes can synthesize several neuropeptides, neurotransmitters, such as SP, calcitonin gene-related peptide (CGRP), ATP, Ach, glutamate, various growth factors, cytokines, chemokines, and many other autacoids, which modulate via corresponding receptors the function of neighbouring neuronal and immune cells [85,94]. After peripheral nerve injury, the synthesis and release of excitatory factors by keratinocytes may be enhanced via Nav activation. Stimulated keratinocytes produce factors, such as SP, CGRP, ATP, and PG that in turn bind to or sensitize receptors on peripheral nerve endings, resulting in depolarization [11,91]. The keratinocytes may express the modulatory analgesic properties as well. For instance, CB2 activation by cannabinoid and noncannabinoid cannabis compounds, such as β-caryophyllene and tetracyclic triterpene euphol, leads to the local release of endogenous opioid β-endorphin from keratinocytes [98,99,100]. The data from clinical and preclinical data confirm the role of keratinocytes in nociception, either in transduction or peripheral modulation of nociceptive input. Theoretically, the receptors and ion channels expressed by keratinocytes and involved in nociception may be targeted by topically administered analgesics, to reduce hyperactivity and release of pronociceptive molecules from skin cells [11,12,13,14,101].

### 2.5. Peripheral NP as the Result of Neuronal and Non-Neuronal Mechanisms

**Figure 2:**
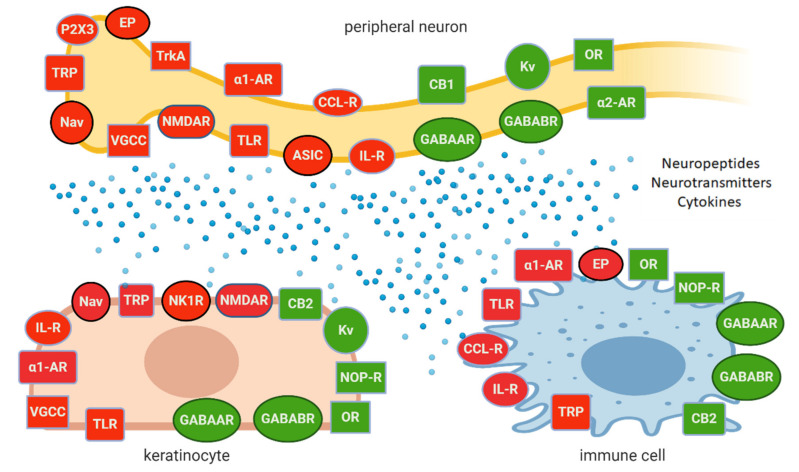
Complex interplay of peripheral neurons, keratinocytes, and immune cells; expressing excitatory (in red) or inhibitory (in green) ion channels or receptors, involved in pain generation, modulation, and maintenance, which potentially may be a target for topical treatments. *Abbreviations:* Nav—voltage-gated sodium channel, TRP—transient receptor potential channel, VGCC—voltage-gated calcium channel, NMDAR—N-methyl-D-aspartate receptor, ASIC—acid-sensing ion channel, TLR—toll-like receptor, α1-AR-α1 adreno receptor, α2-AR—α2 adreno receptor, EP—prostaglandin E2 receptor, GABAAR—gamma-aminobutyric acid receptor A, GABABR—gamma-aminobutyric acid receptor B, Kv—voltage-gated potassium channel, OR—opioid receptor, CB1, CB2—cannabinoid receptor type 1 or 2, NOP-R—nociceptin receptor, CCL-R—chemokine receptor, IL-R—interleukin receptor, TrkA—Tropomyosin receptor kinase A, NK1R—neurokinin 1 receptor. *Created with BioRender.com*.

The functional, structural, and molecular changes induced by peripheral nerve injury occur not only in neurons (both sensory and autonomic) and glial cells, but in non-neuronal cells (keratinocytes; immunocompetent cells—macrophages, mast cells, neutrophils), participating in modulating sensory transduction in the periphery, which have been characterized in several preclinical models [28,56,59,62,64,86,87,101,102,103]. Upon physiological conditions, neuronal and non-neuronal cells in the periphery create a complex interplay on Figure 2, interacting by each other by released neuropeptides, cytokines, and neurotransmitters, acting on corresponding ion channels and receptors. Upon NP conditions, the loop of interactions become overactive and leads to inappropriate sensitivity and functioning of neuronal and non-neuronal cells, resulting finally in hyperexcitability of nociceptors and disturbed signalling from periphery to second-order neurons at spinal level, with several faults either in transduction or transmission. Inappropriate signalling and pain processing result in pain and sensory abnormalities, characteristic for NP such as allodynia and hyperalgesia, which may arise from three neuronal mechanisms [8]:

- peripheral sensitization—hypersensitivity of primary afferent nociceptors;
- central sensitization—increased responsiveness of nociceptive neurons in the CNS to their normal or subthreshold afferent input;
- a switch in the messaging of Aβ fibers from tactile to nociceptive input.

Clinical studies suggest that mechanisms like that observed in preclinical models may also be involved in humans with NP [8]. However, the fundamental question is whether NP, following peripheral nerve injury, is maintained by pathological input from periphery or by central (spinal and supraspinal) generators (centralized pain). It has been confirmed in preclinical trials that hyperexcitable primary afferent neurons (“irritable nociceptors”) and their pathological functioning may induce similar hyperexcitability within the CNS, leading to amplification of incoming peripheral pathological input, maintenance, and aggravation of central sensitization [8,17,104].

Preclinical studies have provided evidence that the early response to peripheral nerve injury in the DRG is driven by macrophages, lymphocytes, and satellite cells, and this is followed by activation of spinal neuronal and glial cells [56]. During the last decade scientists began to formulate new ideas on activated glial cells involvement in synaptic plasticity and signal transduction. This idea is supported by the fact, that neurons and glial cells express on their membranes similar receptors, ion channels, transporters, as well as share similar second messenger systems of intracellular signals. Therefore, pharmacological inhibition of peripheral sensitization can, as consequence, prevent, diminish, and/or cure the central sensitization. The confirmation comes from clinical observations, published by Haroutounian et al. [19] and Gracely et al. [105], who reported reduction in spontaneous pain and allodynia after a peripheral nerve block with lidocaine in patients with NP. Therefore, the data suggests that central sensitization after peripheral nerve injury may be partially maintained by peripheral hyperexcitation and ongoing electrical discharge in sensory neurons. It is reasonable to argue that pathological activity in primary afferent fibres is crucial for NP development [8,19,32,50].

## 3. Topical Administration of Analgesics in LNP

The concept of topical administration of analgesics in LNP emerged from preclinical and clinical trials, where it has been confirmed that:

- input from hyperexcitable primary afferent fibres plays significant role in development and maintenance of NP at the spinal and supraspinal level [8,19,50];
- peripheral neurons exert complex interactions with glial cells, immunocompetent cells, and keratinocytes, contributing to peripheral sensitization and neuronal hyperexcitability on Figure 2 [8,59,86];
- inhibition of peripheral sensitization can diminish and/or eliminate the signs and symptoms of central sensitization [19,105].

Preclinical studies confirmed the role of several ion channels, receptors, enzymes, neurotransmitters, neuropeptides, cytokines and other signalling molecules in peripheral sensitization, inappropriate signalling from periphery and NP behaviour. Evidence for the peripheral mechanisms and their role in NP comes from clinical trials and observations as well, however only a few mechanisms have been directly confirmed in humans:

- role of Nav:
  ◦ in humans with primary erythromelalgia altered firing and ectopic activity in peripheral neurons was observed due to the mutation in gene coding Nav1.7 [36];
  ◦ gain-of-function mutation in Nav1.7, Nav1.8 or Nav1.9 coding genes was associated with small fibre neuropathy and other neuropathic and non-neuropathic pain syndromes [106];
  ◦ loss-of-function mutation in gene coding Nav1.7 or Nav1.9 results in congenital insensitivity to pain [106];
  ◦ increased Nav1.1, Nav1.2, Nav1.5, Nav1.6, Nav1.7 and Nav1.8 expression in the skin of patients with complex regional pain syndrome (CRPS) or postherpetic neuralgia (PHN) [91];
- role of α1-AR—in patients with CRPS α1-AR are upregulated in the epidermis and on dermal nerve fibres [107], activation of α1-AR on human macrophages results in enhanced synthesis of IL-1β [67];
- role of TRPV1—in patients with small fibre neuropathy a statistically significant increase of TRPV1 expression on epidermal keratinocytes was reported [97];
- SNAP-25 (synaptosome-associated protein 25)—plasma membrane protein forming the SNARE (SNAP-receptor), involved in synaptic vesicle fusions, exocytosis, and neurotransmission. SNAP-25 modulates VGCC protein expressed on plasma membrane. Abnormal expression or function of SNAP-25 are observed in chronic pain conditions, including neuropathic pain and fibromyalgia [108].

**Figure 3:**
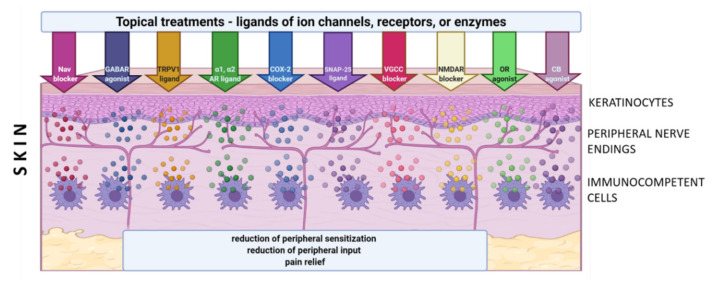
Topical treatments utilized in clinical practice in patients suffering from neuropathic and/or inflammatory pain, and their suggested molecular/cellular targets. *Abbreviations*: Nav—voltage-gated sodium channels, TRPV1—transient receptor potential vanilloid 1, VGCC—voltage-gated calcium channels, NMDAR—N-methyl-D-aspartate receptors, α1-AR—α1 adreno receptors, α2-AR—α2 adreno receptors, GABAR—gamma-aminobutyric acid receptors, OR—opioid receptors, CB- cannabinoid receptors, COX-2—cyclooxygenase 2, SNAP-25—synaptosome-associated protein 25. *Created with BioRender.com.*

Although only a few molecular/cellular mechanisms of NP in humans have been directly confirmed, the preclinical and clinical data support the idea of topical administration of analgesics with different mechanisms of action. Active molecules from topically applied treatments diffuse across the stratum corneum and then penetrate to some extent into the deeper skin layers, where their effect is expected. The molecules of topical drugs act on several distinct ion channels, receptors, or enzymes expressed by either neuronal or non-neuronal cells (Figure 2). The result of this process is the interruption of mutually intensifying stimulation loops, reduction of peripheral sensitization, peripheral input, hyperalgesia, allodynia, and finally reduction of pain intensity in patients with LNP (Figure 3). 

The concept of topical treatments is in line with current theory of mechanism-oriented pain treatment; therefore, they might improve the quality of pain management and patients’ satisfaction with the treatment [9]. According to clinical data, most studied topical treatments (i.e., 5% lidocaine patch, 8% capsaicin patch, BTX-A (botulinum toxin A) in subcutaneous injection) have comparable analgesic efficacy in patients with NP, but relatively few systemic side effects and drug–drug interactions compared to systemic drugs. However, according to systematic reviews, topical drugs still have a weak recommendation for use in patients with LNP [109,110]. In clinical practice, numerous other agents have been used topically in patients with LNP, such as capsaicin at low concentration, antidepressants (amitriptyline, doxepin), antiepileptics (phenytoin, baclofen, gabapentin), ketamine, ambroxol, prazosin, clonidine, opioids (loperamide, morphine), cannabinoids (palmitoylethanolamide, cannabidiol), NSAIDs (nonsteroidal anti-inflammatory drugs) (diclofenac, ibuprofen, ketoprofen), blockers of Nav 1.7 (TV-45070). However, evidence on their efficacy in LNP is inconsistent or inconclusive, therefore they are not included in clinical recommendations [109,110]. In the literature there are numerous preclinical trials pointing out at antinociceptive effect of substances administered topically in animal models of inflammatory and NP. They are not utilized in clinical practice yet and therefore not included in this review [111,112]. Table 1 and Table 2 present the most studied and suggested mechanisms of action of topical treatments, being used in clinical practice. 

Topical drugs utilized in subjects with LNP exert multiple mechanisms of action, but which ones are most crucial and responsible for analgesic effect observed in humans has not been fully elucidated. Given that interactions between neuronal and non-neuronal cells involve multiple mediators and broad spectrum of receptors, single agents targeting multiple mechanisms or combination of agents targeting single mechanisms might be particularly useful in clinical settings [11,12,13,14]. In the subsequent sections, a brief discussion regarding data covered in Table 1 and Table 2 is provided.

### 3.1. Treatments Acting on Voltage-Gated Sodium Channels

The role of Nav in physiological nociception and different pain states has been confirmed in numerous preclinical studies [37,38,71] and several clinical observations [36,91,106], therefore substances blocking Nav receive special attention in pain medicine in humans [113]. 

Topically applied lidocaine, phenytoin, antidepressants (amitriptyline, doxepin) and ambroxol are thought to exert their antinociceptive effect in patients with LNP mainly via Nav inhibition [114,115,116,117,118,119,120,121]. In clinical practice, topical treatments acting on Nav provide beneficial analgesic effect; however, 5% lidocaine patches only have scientific evidence sufficient to position this treatment in clinical recommendations for NP management [109,110].

In clinical studies conducted in patients with PHN specific blocker of Nav1.7 (TV-45070) has been assessed as well, but no statistical difference was observed between active treatment and placebo for the change in mean daily pain scores from baseline compared with the last week [122].

Other substances being suggested to exert their antinociceptive effect via Nav blockade in vitro in peripheral nerves include NSAIDs, opioids, α2-AR agonists, and plant-derived compounds, which has been extensively reviewed by Kumamoto [123].

### 3.2. Treatments Acting on Transient Receptor Potential Vanilloid 1 Channels

Some members of the TRP family deserve special attention in pain medicine, as they are expressed in nociceptors, play crucial role in physiological nociception (TRPV1-4, TRPM8, and TRPA1), and are also involved in the generation and maintenance of chronic pain [69,70,76,93,94,124]. The available data suggest that, in particular, TRPV1 expressed by C-fibers nociceptors may play an important role in nociception and in pathomechanism of neuropathic and inflammatory pain [69,70,76,93,94,97,125]. Moreover, TRPV1 channels are widely distributed in peripheral and central nervous systems, and in other non-neuronal cells involved in peripheral nociception such as keratinocytes and immune cells [125].

Capsaicin is a highly selective agonist of the TRPV1 channels utilized in clinical setting either in low (<0.1%) or in high (8% patches) concentration [126]. Clinical evidence supports only 8% capsaicin patches in patients with LNP [109,110,127], whereas the evidence for the low concentration capsaicin is inconclusive [109,110]. Although capsaicin is the potent agonist of TRPV1, its long term analgesic effect relies on the massive intracellular influx of ions (Ca^2+^, Cl^−^) following activation of TRPV1 and subsequent damage of the cytoskeleton and mitochondria. This leads to the defunctionalisation of hyperexcitable nociceptive receptors, or a temporary destruction of peripheral nerve endings [125].

Other drugs acting via TRPV1 include NSAIDs such as diclofenac, ketorolac, xefocam, which, applied topically in rats inhibited pain behaviour, most probably by inhibition of TRPV1 and TRPA1 channels [128].

### 3.3. Treatments Acting on Voltage-Gated Calcium Channels

VGCCs are widely distributed in neuronal and non-neuronal cells. Studies confirmed expression of L-type calcium channel in excitable cells [129] and in epidermal keratinocytes, where they play role in skin barrier homeostasis [96]. In turn the activity of T-type calcium channels is increased in NP states, such as traumatic nerve injury, peripheral diabetic neuropathy or CIPN (chemotherapy-induced peripheral neuropathy) [39,61,76,91,94,130,131].

In clinical settings, VGCC blocker gabapentin administered orally is commonly used and recommended as the first line treatment in patients with NP [109,110]. Single observational studies and case reports report beneficial analgesic effect in patients with LNP states after topical administration of cream containing gabapentin, but the scientific evidence is inconclusive [132].

Other drugs possibly acting via VGCC blockade include lidocaine, however in vitro the VGCC blockade has been observed in lidocaine concentrations 100-fold higher than needed for Nav blockade [133].

### 3.4. Treatments Acting on N-Methyl-D-aspartate Receptors

The role of neuronal NMDAR in generation of peripheral neuroinflammation and central sensitization has been confirmed in several preclinical and clinical studies [134,135,136]. Moreover, the experimental studies confirmed the expression of functionally active NMDAR in keratinocytes in human normal and inflamed skin, where they play a role in epidermal homeostasis and nociception [95,137].

Ketamine, an anaesthetic drug, is suggested to act topically by blockade of NMDAR and subsequent inhibition of glutamate release [136,138]. In clinical trials topical ketamine is more commonly used in combination with other drugs, showing beneficial analgesic effect in patients with different NP syndromes [139], however human studies on topical ketamine as a single agent are inconsistent [140].

Other drugs with possible antinociceptive effect via NMDAR blockade include antidepressants (i.e., amitriptyline)—but this effect has been observed in cultured rat brain neurons only [141]—and diclofenac, providing antinociceptive effect after topical administration in rats [142].

### 3.5. Treatments Acting on α1 Adrenergic Receptors

The role of autonomic system and adrenergic receptors in generation and maintenance of NP has been confirmed [31,33,43,44,45,46,67,73]. Upregulated α1-AR in peripheral neurons, keratinocytes, and immune cells may be targeted by prazosin, an antagonist of α1-AR [143]. Topically administered prazosin has been studied in one study in healthy volunteers and patients with CRPS to date, showing analgesic effect [144]. 

Other drugs with possible antinociceptive effect related to α1-AR blockade are antidepressants such as nortriptyline, imipramine, maprotiline, and milnacipran. Their antinociceptive effect via AR blockade has been observed after systemic administration in formalin test only [145]. However, whether amitriptyline acts via α1-AR blockade is unclear.

### 3.6. Treatments Acting on Cyclooxygenase 2

After peripheral nerve injury, PGs may be synthetized not only in invaded immune cells, but in neuronal and glial cells as well. PGs regulates the function of peripheral sensory nerves in paracrine and autocrine manners in several models of NP [75]. Topically administered NSAIDs may interfere with the proinflammatory and pronociceptive effects of PGs by their ability to inhibit the cyclooxygenase COX-2.

In clinical studies, gel containing 1.5% diclofenac gave satisfactory pain relief in patients with NP syndromes, however the evidence was medium [146]. Topical NSAIDs are not widely used in patients with NP, rather recommended and commonly used in pain syndromes with predominant inflammatory mechanism [147].

### 3.7. Treatments Acting on Synaptosome-Associated Protein 25

Among the topical analgesics in NP, BTX-A deserves special attention, because when given topically in the periphery, it can directly modulate both central and peripheral sensitization. Recent animal studies proved that topical BTX-A administration is possibly followed by the retrograde transport and transcytosis, which are responsible for pain relief [148,149,150,151].

In clinical practice, local injections of BTX-A are recommended as the third-line treatment in patients with LNP, but scientific evidence for its use is weak [109,110].

### 3.8. Treatments Acting on Gamma-Aminobutyric Acid Receptors

GABA is the major inhibitory neurotransmitter in the adult mammalian central nervous system and exerts inhibitory action via specific receptors named GABAAR and GABABR. GABAAR are ion channels located in postsynaptic membranes, whereas GABABR are GPCR located both in pre- and postsynaptic membranes [152]. Moreover, GABAR are expressed in immune and skin cells, where they are involved in skin barrier homeostasis and skin inflammatory diseases [83,84,153]. In preclinical studies, the role of peripheral GABABR in nociception has been confirmed in animal model of allodynia [154].

In clinical settings, baclofen, a specific agonist of GABABR, is utilized topically in patients with NP syndromes in a mono- or add-on therapy, but the evidence of its efficacy is inconclusive [155,156].

Other drugs possibly acting by GABA receptors include:

- antidepressants (amitriptylline, fluoxetine), but their antinociceptive effect has been observed after intraperitoneal administration in rats [157];
- ketamine—the agonist to GABAAR, which has been confirmed in an anesthetic model in mice [158];
- phenytoin, which potentiated GABA-induced currents in cultured rat cortical neurons through modulation of the GABAAR [159].

### 3.9. Treatments Acting on α2 Adreno Receptors

The α2-AR are inhibitory G-protein coupled receptors involved in nociception and expressed in peripheral and central nervous system [160,161,162]. Activation of these receptors induces antinociception in animal models of NP [160] and reduced production of TNFα, IL-6, IL-8 in in vitro studies [163].

Clonidine, an agonist for α2-AR receptors, is an extremely potent antinociceptive agent when given systemically. However, topical clonidine exerts an analgesic effect in patients with LNP with medium level of evidence [164].

### 3.10. Treatments Acting on Opioid Receptors

The peripheral inflammation increases de novo synthesis of OR in DRG and their density in the peripheral nerve endings, whereas nerve injury decreases MOR (μ opioid receptor) expression in peripheral nerves [165]. However, peripheral nerve injury via cytokines, especially chemokines and other factors recruits immune cells to the site of injury. Moreover, recruited immune cells release endogenous opioids and express all types of OR [79,166]. Besides, OR are expressed as well by keratinocytes, which can produce β-endorphins [167]. Taken together, following peripheral injury, OR expressed by neuronal and non-neuronal cells and their endogenous agonists form a complex system, modulating nociception at the peripheral level [82]. Therefore, there is a rationale for topical use of opioids, without involvement of central mechanism of action, which has been confirmed in NP models [80,168].

In clinical practice, loperamide has been used in patients with NP, however evidence is limited to single case report only [169]. To date, morphine in topical formulation showed beneficial effect in patients with painful mucosal or skin lesions due to cancer [170], but the data on topical morphine in NP is lacking.

### 3.11. Treatments Acting on Cannabinoid Receptors

Numerous studies indicate a modulatory effect of the endocannabinoid system in NP [171]. In the periphery, CB1 receptors are expressed on nociceptive nerve endings and the DRG, whereas CB2 receptors are located in immune cells and keratinocytes [78,172]. Either CB1 or CB2 receptors may be targeted by cannabinoids administered topically, evoking analgesic effect in both inflammatory and neuropathic pain [78,81,98,99,100,172].

Clinical observations indicate that topical administration of CBD (cannabidiol), mixed with other well-known anti-inflammatory phytoderived products, exert analgesic and anti-inflammatory effect, but evidence is weak and comes from single studies on inflammatory pain only [173]. In patients with vulvodynia, beneficial analgesic effect of topically administered 1% PAE (palmitoylethanolamide), an endocannabinoid anti-inflammatory compound, with 5% baclofen has been observed [174]. The clinical observations support the topical application of cannabinoids, but data and evidence on their efficacy in LNP syndromes are lacking.

## 4. Conclusions

The progress made in identification of peripheral mechanisms of NP, peripheral neuronal and non-neuronal cells interplay, and the role of peripheral sensitization in modulation of central hypersensitivity has given a stronger rational basis for topical treatments in clinical practice and experimental research on novel agents. It is accepted that primary sensory afferent neurons, immune cells, and keratinocytes express numerous ion channels and receptors, release signaling molecules in a response of injury, and can be activated or suppressed by a wide range of pro- or antinociceptive mediators, respectively. Modulation of their complex interactions in the periphery represents a strategy for the development of new topical analgesics and their utilization in clinical settings.

## Figures and Tables

**Table 1:** Receptors, ion channels, enzymes with excitatory mode of action, involved in generation and maintenance of NP, potentially targeted by topically administered treatments. Possible site of action of topical agents, addressing given molecular target. *Abbreviations:* Nav—voltage-gated sodium channels, TRPV1—transient receptor potential vanilloid 1, VGCC—voltage-gated calcium channels, NMDAR—N-methyl-D-aspartate receptors, α1-AR—α1adreno receptors, COX-2—cyclooxygenase-2, NSAID—nonsteroidal anti-inflammatory drug, SNAP-25—synaptosome-associated protein 25.

	Receptor Ion Channel Enzyme	Topical Agent Utilized in Clinical Practice	Possible Site of Action	Reference
EXCITATORY	Nav	Lidocaine Antidepressants: -Amitriptyline -Doxepin Phenytoin Ambroxol TV-45070 Opioids NSAIDs Clonidine	Neurons Keratinocytes	[36,37,38,91,106,114,115,116,117,118,119,120,121,122,123]
	TRPV1	Capsaicin NSAIDs	Neurons Keratinocytes Immune cells	[69,70,76,93,94,97,124,125,126,127,128]
	VGCC	Gabapentin Lidocaine	Neurons Keratinocytes	[39,61,76,91,94,96,129,130,131,132,133]
	NMDAR	Ketamine Antidepressants: -amitriptyline NSAID-Diclofenac	Neurons Keratinocytes Immune cells	[95,134,135,136,137,136,137,138,139,140,141,142]
	α1-AR	Prazosin Antidepressants	Neurons Keratinocytes Immune cells	[31,33,43,44,45,46,67,73,143,144,145]
	COX-2	NSAIDs	Neurons Immune cells Schwann cells	[75,146,147]
	SNAP-25	Botulinum toxin A	Neurons Immune cells Glial cells	[148,149,150,151]

**Table 2:** Receptors, ion channels with inhibitory mode of action, involved in modulation of NP, potentially targeted by topically administered treatments. Possible site of action of topical agents, addressing given molecular target. *Abbreviations:* GABAR—gamma-aminobutyric acid receptors, GABAAR—gamma-aminobutyric acid receptors A, GABABR—gamma-aminobutyric acid receptors B, α2-AR—α2 adreno receptors, OR—opioid receptors, CB—cannabinoid receptors.

	Receptor Ion Channel	Topical Agent Utilized in Clinical Practice	Possible Site of Action	Reference
INHIBITORY	GABAR	Antidepressants: Amitriptyline	Neurons Keratinocytes Immune cells	[83,84,152,153,157]
	GABAAR	Ketamine Phenytoin		[158,159]
	GABABR	Baclofen		[154,155,156]
	α2-AR	Clonidine	Neurons	[160,161,162,163,164]
	OR	Opioids	Neurons Keratinocytes Immune cells	[79,80,82,165,166,167,168,169,170]
	CB	Cannabinoids	Neurons Keratinocytes Immune cells	[78,81,98,99,100,171,172,173,174]
